# N^6^ -Methyladenosine Modification in Chronic Stress Response Due to Social Hierarchy Positioning of Mice

**DOI:** 10.3389/fcell.2021.705986

**Published:** 2021-08-20

**Authors:** Malena dos Santos Guilherme, Theodora Tsoutsouli, Hristo Todorov, Sina Teifel, Vu Thu Thuy Nguyen, Susanne Gerber, Kristina Endres

**Affiliations:** ^1^Department of Psychiatry and Psychotherapy, University Medical Center, Johannes Gutenberg University Mainz, Mainz, Germany; ^2^Institute of Human Genetics, University Medical Center, Johannes Gutenberg University Mainz, Mainz, Germany

**Keywords:** dominance, behavior, epigenetic modification, methyltransferase, transcriptomics, sex difference

## Abstract

Appropriately responding to stressful events is essential for maintaining health and well-being of any organism. Concerning social stress, the response is not always as straightforward as reacting to physical stressors, e.g., extreme heat, and thus has to be balanced subtly. Particularly, regulatory mechanisms contributing to gaining resilience in the face of mild social stress are not fully deciphered yet. We employed an intrinsic social hierarchy stress paradigm in mice of both sexes to identify critical factors for potential coping strategies. While global transcriptomic changes could not be observed in male mice, several genes previously reported to be involved in synaptic plasticity, learning, and anxiety-like behavior were differentially regulated in female mice. Moreover, changes in N^6^-methyladenosine (m^6^A)-modification of mRNA occurred associated with corticosterone level in both sexes with, e.g., increased global amount in submissive female mice. In accordance with this, METTL14 and WTAP, subunits of the methyltransferase complex, showed elevated levels in submissive female mice. N^6^-adenosyl-methylation is the most prominent type of mRNA methylation and plays a crucial role in processes such as metabolism, but also response to physical stress. Our findings underpin its essential role by also providing a link to social stress evoked by hierarchy building within same-sex groups. As recently, search for small molecule modifiers for the respective class of RNA modifying enzymes has started, this might even lead to new therapeutic approaches against stress disorders.

## Introduction

Transcriptional changes in various brain regions have been reported in both, rodent models of depression and depressed humans (e.g., Guilloux et al., 2012; Vialou et al., 2014; Bagot et al., 2015). In the commonly used chronic social defeat (CSD) paradigm, male mice are exposed repeatedly to a bodily superior cage-resident and kept afterward in sight and odorant reception contact to these (Krishnan et al., 2007; Krishnan and Nestler, 2008). Subsequently, analysis of social behavior after the stress exposure period facilitates the classification of mice as resilient (remaining social interest) and susceptible (avoiding social interaction with a mouse similar to the aggressor). Using an integrative approach, networks of co-regulated genes associated with mental susceptibility or resilience were already identified in the aforementioned brain regions (Bagot et al., 2016). For example, Dkkl1 and Neurod2 were found to control stress susceptibility when overexpressed. However, even though mouse models of CSD stress have been comprehensively studied in male mice, one point of critique is the dominating component of physical attack that can further lead to injuries and wounding and thereby evoke neuro-immunological consequences. In females, adaptation of the male CSD paradigm can still include the physical component [pheromone-based or chemogenetic approaches (Takahashi et al., 2017; Harris et al., 2018)]. However, models applicable to both sexes with reduced physical contact time or witness approaches, where the stress is reduced to the mere mental component, are of high interest (Goto and Toyoda, 2015; Sial et al., 2016). The vicarious social defeat stress model, for example, results in behavioral deficits such as social avoidance and other depressive- and anxiety-like phenotypes and evokes a corticosterone spike without physical injury of the to be tested adult male mouse (Sial et al., 2016). Similar results were also observed for female mice witnessing the attack of an aggressor against a male conspecific (Iniguez et al., 2018).

Another option to investigate chronic social stress might lie in using the intrinsic social hierarchy of cage-mates, where mild physical attack and chasing continuously occur (So et al., 2015) but seldomly result in severe wounding. Social hierarchies in free-living animals are likely to emerge due to food restriction, access to mating partners, or territory. However, male social hierarchies are very common as inter-sexual competition and have repeatedly been reported in group-housed laboratory mice (Haemisch et al., 1994; Howerton et al., 2008; Stagkourakis et al., 2018). Recently, the establishment of hierarchies in outbred CD-1 virgin female mice has similarly been characterized (Williamson et al., 2019), even if less pronounced than in males. These hierarchical structures, together with unique factors such as intra-uterine position [reviewed in Lathe (2004)], can impact behavior comparable to pharmacological treatments. For instance, overall activity was reported to be increased in dominant male mice (D’Amato, 1988; Hilakivi-Clarke and Lister, 1992) while alcohol abuse was more frequently described in rats with low social status (Ellison and Potthoff, 1984). One explanation for the observed changes might rely on different activation of the HPA axis: dominant male rodents mostly show elevated stress hormone levels such as corticosterone in serum (Williamson et al., 2017). Moreover, glucocorticoid receptor mRNA was shown to be decreased in the hippocampal CA1 region by *in situ* hybridization of female co-housed subordinate rats (Chao et al., 1993). Another example is the higher mRNA expression of CRF in the medial amygdala and hypothalamus of more dominant outbred CD1 male mice (So et al., 2015). However, for C57BL/6NCrSlc (B6), an increased hypothalamic CRF mRNA level was reported for submissive mice, probably hinting at strain-specific differences regarding transcriptional changes (Horii et al., 2017).

Regulation of gene expression in response to stressful experiences also involves epigenetic mechanisms such as DNA methylation and chromatin modifications (de Kloet et al., 2005; McEwen et al., 2015; Stenz et al., 2018). More recently, N^6^-methyladenosine (m^6^A) mRNA modification was identified as part of the stress-evoked epitranscriptome. m^6^A is a major internal mRNA modification present in at least 25% of all RNAs (Dominissini et al., 2012; Meyer et al., 2012; Linder et al., 2015). It plays a crucial role in several cellular mechanisms such as heat shock-response, DNA-damage response, or developmental processes (Meyer et al., 2012; Zhou et al., 2015; Xiang et al., 2017). During transcription, m^6^A is attached (Ke et al., 2017; Slobodin et al., 2017) by a methyltransferase complex consisting of multiple proteins including METTL3, METTL14 (methyltransferase-like 3/14) (Liu et al., 2014), and WTAP (Wilms’ tumor 1-associated protein) (Ping et al., 2014). It can be removed from mRNA by the demethylases FTO (fat mass and obesity-associated factor) (Jia et al., 2011; Mauer et al., 2017) and ALKBH5 (AlkB homolog 5) (Zheng et al., 2013). The m^6^A modification can contribute to both, either transcript stabilization or mRNA decay (Mauer et al., 2017). The ultimate effect of the RNA modification is exerted by the associated readers and context-specificity of the respective modification site: for example YTHDF2 presents a decay-inducing reader, while FMRP competes with this protein and thereby enhances mRNA stability [reviewed in Dermentzaki and Lotti (2020) and He and He (2021)]. By applying 15 min restrain stress in adult C57BL/6 male mice, Engel et al. (2018) demonstrated for the first time that this exposure as well as corticosterone (CORT) administration alters m^6^A modification levels and the corresponding enzymatic apparatus region- and time-specifically in the brain.

In this study, we aimed at elucidating the global effects of intrinsic social stress due to social rank position on the regulation of gene expression in the brain. Therefore, we identified dominant and submissive mice with the classical tube test in male and female animals and measured transcriptional changes with RNA-seq analysis and m^6^A levels within mRNA by an antibody-based assay. Additionally, we assessed changes in the enzymes involved in m^6^A modification of mRNA.

## Materials and Methods

### Animals

C57BL/6JOlaHsd mice (male and female) were obtained at the age of 3 weeks (Envigo) and were group-housed with four animals per cage in the animal facility of the Department of Psychiatry and Psychotherapy (JGU Mainz). At the age of 8 weeks animals were subjected to short-term handling and weighing procedure to prevent experimenter-driven stress in the tube test or during the sacrifice. The room was kept on a 12/12 light/dark cycle, with white light on at 6:00 a.m. Food and water were provided *ad libitum*. All animal experiments were carried out in accordance with the recommendations of the European Communities Council Directive regarding care and use of animals for experimental procedures and were approved by local authorities (Landesuntersuchungsamt Rhineland-Palatinate; approval number G17-1-035).

### Tube Test

The tube test was established to investigate dominance in different mouse strains but can also be applied in rats. It combines the advantage of a simple apparatus with minimized physical injuries [reviewed in Fan et al. (2019)]. For C57BL/6 mice, in general, a good correlation with other dominance measures such as food competition or fighting has been observed.

**FIGURE 1:**
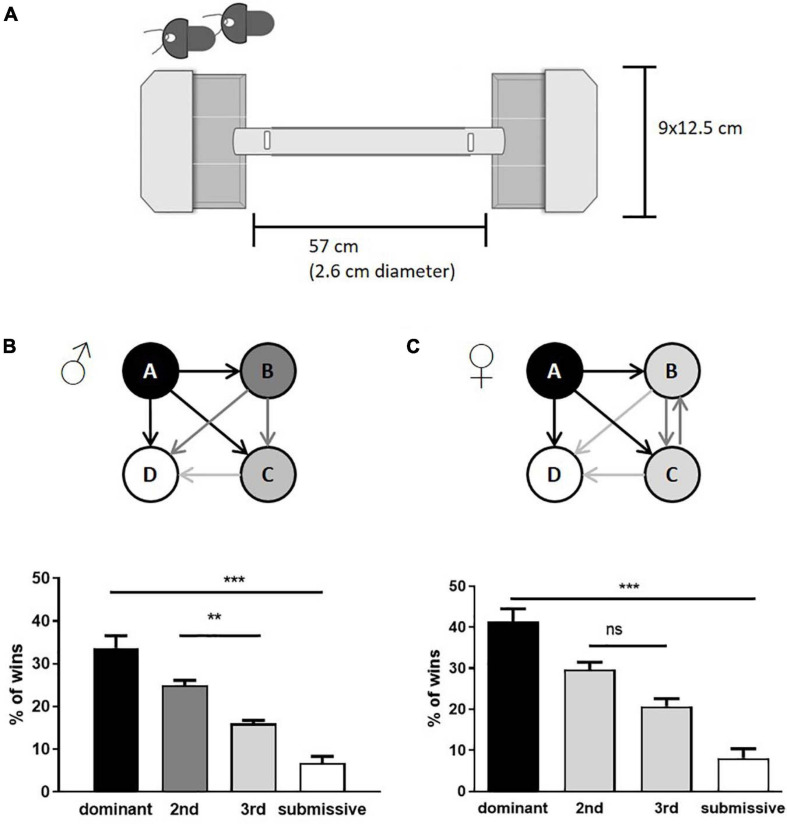
Assessment of social hierarchy in group-housed male and female mice. Schematic representation of tube test equipment is shown in **(A)**. The test consisted of two non-transparent boxes with lids where the mice were placed. At the same time, access to the tube was denied by inserted shutters (indicated in the upper left corner, the shutter position within the closed setup as labelled by vertical white rectangles). The boxes were subdivided with transparent plastic sheets, limiting the freedom of movement, and motivating the mice to enter the tube. Both shutters were removed simultaneously, and the confrontation of mice was observed within the transparent tube. Cage hierarchies were identified by the number of test runs considered as “win” per mouse (male: **B**; female: **C**). Arrows indicate the mean of relations within the cage groups; the number of wins in % of dominant and submissive animals is given as mean + SEM. Statistical analysis was performed with a one-way ANOVA with Bonferroni’s post-hoc comparison test [*n* = 28 mice per sex in total were tested, in each category (e.g., “dominant”) all test values from the seven mice belonging to this category are integrated; **p < 0.01, ***p < 0.001; ns, *p* > 0.05].

At the age of 10–12 weeks, animals were subjected to the tube test to assess their dominance score. 28 male mice and 20 female mice were investigated accordingly. The apparatus consisted of a colorless acrylic glass tube (57 cm length; 2.6 cm diameter) connected at each end to a pipette tips box (12.5 cm × 9 cm), which served as starting boxes for the mice. Small slots (3 cm from both ends of the tube) were used to insert cardboard pieces to partition the entrances to the tube, allowing pulling them simultaneously from a distance to start the individual test run (see, Figure 1A). Mice received a 3-day habituation procedure where they were allowed to explore the apparatus three times. A Fruit Loop^TM^ [KELLOGG (Deutschland) GmbH, Hamburg, Germany] was placed in the box opposite to the start point for a positive incentive.

During the social hierarchy trials, mice from the same home cage competed against each other to identify dominant and submissive members of a cage. All possible combinations were tested three times. A mouse was considered the “winner of the trial” as soon as it pushed the opponent entirely back into its starting box. Following every trial, the apparatus was cleaned with an alcohol solution. Test runs without clear winners were repeated once. Female mice identified as intermediates were mixed into new groups of four and tested after 1 week again for their social status (*n* = 8). All mice were sacrificed 3–4 days after assessment of their social status in the morning (9:00 a.m.–12:00 p.m.).

### Tissue Preparation

The dominant and submissive mice of each cage (*n* = 7 each) were sacrificed simultaneously by decapitation after isoflurane anesthesia to avoid social instability if e.g., only the dominant animal would be separated from its group. Spleen, thymus, adrenal glands, both brain hemispheres, and blood were collected. The right brain hemispheres, chopped into three pieces each, were immediately placed in RNAlater (Qiagen) for stabilization before RNA-seq, all other samples were shock-frozen in liquid nitrogen. All samples were stored at −80°C until further use. Serum was obtained by centrifugation of the blood samples (1650 *g*, 10 min, 10°C, and 15680 *g*, 10 min, 10°C).

### Analysis of Serum Corticosterone by HPLC

Serum samples were collected as described before, and CORT quantification was conducted by adapting the protocols of Wong et al. (1994) and Viljoen et al. (2012). In brief, 45 μl serum were mixed with dexamethasone (500 ng/ml) (Cayman Chemicals, Ann Arbor, MI, United States) as an internal standard and a fixed amount of CORT to facilitate measurement of the endogenous levels (200 ng/ml) (Sigma-Aldrich, St. Louis, MO, United States). Samples and standards (duplicates, 50–750 ng/ml) were extracted two times by adding diethyl ether (1:2, v:v, Thermo Fisher Scientific, Hampton, NH, United States), mixing at RT for 10 min, and centrifugation for 10 min, 3800 *g*, 4°C. The organic phases were combined and evaporated at 40°C. The residue was reconstituted in 65 μl mobile phase (water and acetonitrile, 65/35, v/v, acetic glacial acid to adjust the pH to 4.1–4.2), and 50 μl were subjected to reversed-phase HPLC analysis. The HPLC (Automatic liquid autosampler and isocratic pump: Series 1100; thermostatted column compartment and variable wavelength detector: Series 1200, all Agilent Technologies, Waldbronn, Germany) was equipped with an ODS Hypersil C_18_ column (5 μm, 150 mm × 4 mm; MZ-Analysentechnik GmbH, Mainz, Germany) tempered at 24°C. Detection was carried out at 245 nm with a consistent flow rate (1.2 ml/min). Chromatograms were analyzed by ChemStation for LC software (Rev.A.10.02, Agilent Technologies, Waldbronn, Germany). To exclude effects evoked by circadian rhythm of corticosterone (e.g., Fonken and Nelson, 2014), data were analyzed pairwise in technical duplicates and the respective value of the dominant animal used as reference for the corresponding submissive animal serum specimen.

### RNA Preparation

For extraction of RNA from the right hemisphere (stored in RNAlater at −80°C), the RNeasy Lipid Tissue Mini Kit (Qiagen) was used following the instructions of the vendor and including the optional DNAse on-column digestion (*n* = 7 for males and 4 for females per group).

### Library Preparation and RNA Sequencing

Total RNA sequencing was employed to study the expression profile of brain samples (right hemisphere) from male and female mice with respect to social rank-specific changes. The extracted RNA was sequenced at the Institute of Molecular Biology in Mainz, Germany. The Illumina TruSeq stranded total RNA Human/Mouse/Rat kit was used for library preparation containing RiboZero beads for rRNA depletion from total RNA. RNA integrity was measured prior to sequencing and all samples had RIN values above 8.2 (mean 8.78 ± 0.25). Sequencing was realized with the Illumina NextSeq 500 platform, and the High Output v2 kit with an average output of 32 million single-end (85 nt) reads per sample.

### Bioinformatic Analysis of RNA-Seq Data

Raw sequencing files in fastq format were first inspected with FastQC v0.11.8 for quality control. Adapter trimming was performed with cutadapt v1.15. Afterward, quality trimming was conducted with bbduk.sh v38.06 from the BBMap suite with a cutoff of Q10. The trimmed and cleaned reads were mapped to the reference genome of mm10 using the STAR aligner v2.6.0a (Dobin et al., 2013) with default settings. The annotation files were downloaded from the Illumina iGenomes website. The percentage of uniquely mapped reads varied between 82.6 and 86.7% (mean 84.4 ± 1.11%). After quality checks, a count matrix was created using featureCounts v1.6.2 (Liao et al., 2014). Differential expression analysis was performed using the DESeq2 R package v1.1.383 (Love et al., 2014). The significance of log2-fold changes was evaluated by Wald *z* tests. The threshold for differentially expressed genes (DEG) was set to an adjusted *p*-value < 0.05. Control for the false discovery rate was executed with the Benjamini–Hochberg method. Changes in the overall expression pattern between experimental groups were investigated with a principal component analysis (PCA) using the top 500 most variable genes. Heatmaps of rlog transformed counts were produced with the pheatmap function. The raw sequencing data from this study are available at GEO under the accession number GSE161198.

### qPCR

For qPCR, the RNA concentration was adjusted to 50 ng/μl. The expression of Mettl14 and Wtap was analyzed using the QuantiTect^®^ SYBR^®^ Green RT-PCR Kit (Qiagen) following the manufacturer’s instructions using 100 ng RNA per reaction and the respective QuantiTect Primer Assay (Qiagen). GAPDH mRNA levels served as a housekeeping control. For the analysis, 4–6 samples per group were used.

### m^6^A-mRNA Methylation Assay

To assess the m^6^A methylation status of mRNA isolated from the brain, the EpiQuick^TM^ m^6^A RNA Methylation Quantification Kit (EpiGentek) was used following the manufacturers’ recommendations. The assay for a single point control was conducted using 200 ng RNA per sample. The assay’s specificity was proven by analyzing poly-A+ RNA samples from either wild type *Drosophila melanogaster* or knock-out animals for METTL3 (see Supplementary Figure 1).

### Western Blotting

For Western blotting and related experiments, nuclear fractions of left brain hemispheres were extracted using the Nuclear Extract Kit (Active Motif Europe, La Hulpe, Belgium) following the vendor’s manual to extract frozen tissue. Protein content was quantified using Roti-Nanoquant (Carl Roth, Karlsruhe, Germany), and aliquots of nuclear extracts were stored at −80°C until further use. Protein concentration was adjusted to 20 μg/10 μl in LDS NuPAGE buffer (1x, Life Technologies, Carlsbad, CA, United States) with DTT (1 M, 10% v/v) and boiled for 5 min, 95°C. Proteins were separated on a 10% SDS-polyacrylamide gel, transferred onto nitrocellulose membranes (GE Healthcare, Chicago, IL, United States) and stained with Ponceau S red (0.2% in 1% acetic acid, Carl Roth, Karlsruhe, Germany) for absolute protein quantification. After destaining with PBS, membranes were cut in appropriate sections and blocked in 5% milk buffer (Carl Roth, Karlsruhe, Germany), containing 0.1% Tween 20 (AppliChem GmbH, Darmstadt, Germany) in TBS (for an exemplary uncropped blot and the respective Ponceau S red stain, see Supplementary Figure 2). Primary antibody incubation took place overnight at 4°C. Antibodies against METTL3, METTL14, WTAP, or Virilizer [all antibodies are part of the N^6^mA methyltransferase antibody sampler kit: METTL3 (D2I6O) rabbit mAb 96391; METTL14 (D8K8W) rabbit mAb 51104; Virilizer (D4N8B) rabbit mAb 88358, WTAP antibody 56501; Cell Signaling Technology, Danvers, MA, United States] were applied in a dilution of 1:1000 in 5% BSA (Carl Roth, Karlsruhe, Germany), 0.1% Tween 20 in TBS or 5% milk, 0.1% Tween 20 in TBS (for WTAP antibody). After adding the secondary horseradish peroxidase-coupled anti-rabbit antibody (dilution 1:3000), blots were developed by using SuperSignal West Femto ECL reagent (Thermo Fisher Scientific, Waltham, MA, United States) and signals detected by using a CCD camera imaging system (Stella camera, Raytest Isotopenmessgeräte GmbH, Straubenhardt, Germany). The densitometric analysis was performed with the image analysis software AIDA (Version 4.26, Raytest, Straubenhardt, Germany).

### FTO/ALKBH5 Demethylase Activity Assay

Demethylase activity in nuclear extracts derived from brain hemispheres (see above) was measured using the Epigenase m^6^A Demethylase Activity/Inhibition Assay Kit (EpiGentek, Farmingdale, NY, United States) by following the manufacturer’s instructions. The assay was performed using 12.5 μg of protein per sample.

### Methyltransferase Activity Assay

Methyltransferase activity was measured by using the MTase-Glo Methyltransferase assay (Promega, Madison, WI, United States). To narrow the specificity toward the METTL3/METTL14 methyltransferase complex, polyA RNA served as a substrate (20mer, 200 nM, Eurofins Scientific, Hamburg, Germany). 1 μl of nuclear extracts (equivalent to 4–6 μg protein) were used, whereby each sample was measured without addition of S-adenosylmethionine (SAM) to account for endogenous concentrations of S-adenosylhomocysteine (SAH), ADP, and ATP. These background values were subtracted from values obtained by full supplemented reactions (with SAM) and normalized to protein content. Values were calculated as the percentage of the mean value obtained for dominant individuals.

### Statistical Analysis

All statistical analyses were performed using GraphPad Prism 6 or 8 for Windows or R version 3.5. Data are graphically presented as mean + standard error of the mean (SEM). Statistical analysis was performed with an unpaired, two-tailed Student’s *t*-test if not stated otherwise (^∗^*p* < 0.05; ^∗∗^*p* < 0.01; ^∗∗∗^*p* < 0.001).

## Results

### Identification of Dominant and Submissive Individuals via the Tube Test in Group-Housed Mice

The tube test is a well-established paradigm to investigate social hierarchies in group-housed mice and has furthermore been used to study mental diseases or genotypes (Lindzey et al., 1961; Yang et al., 2015). Although both, male and female animals, form social hierarchies in groups, males have been preferred in experiments across the literature (Yang et al., 2015; McNamara et al., 2018; Nakata et al., 2018). We used male and female C57BL/6JOlaHsd mice at the age of 10–12 weeks, housed in groups of four. After individual habituation to the setup, the tube test was conducted (see Figure 1A for the experimental setup) with all possible combinations of individuals. As expected, male mice built a strict social hierarchy with defined intermediates as described previously (van den Berg et al., 2015). On the contrary, only dominant and submissive mice could be identified within groups of females, while the two intermediates were not discriminable (Figures 1B,C). In both sexes, dominant animals showed an average of over 30–40% of total wins while submissive individuals won less than 10% of the runs.

**FIGURE 2:**
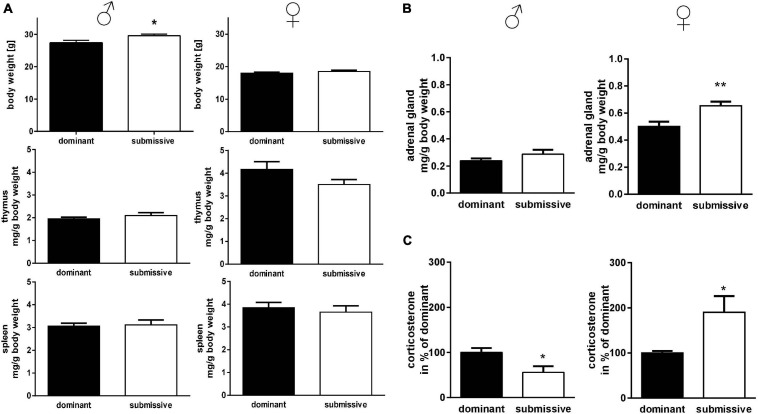
Physical parameters in accordance with social status. Body weights of mice identified as dominant or submissive as well as thymus and spleen weights were assessed **(A)**. Adrenal glands were weighed (sum of both glands) **(B)** and CORT concentration of serum samples was measured by HPLC analysis **(C)**. Data are presented as mean + SEM. Statistical analysis was performed with unpaired Student’s *t*-test. Sample size was (*n* = 7) per group except for dominant female spleen weight (*n* = 6) and adrenal gland weight of submissive males (*n* = 6). The lacking data points were removed due to incomplete dissection and thereby non-plausible tissue weight. Statistical analysis was performed with unpaired Student’s *t*-test (**p < 0.01; *p < 0.05).

To investigate potential differences in mice’s lymphatic system with different social positioning, we weighed the thymus and spleen: interestingly, submissive male mice displayed a significant increase in body weight compared to their dominant counterparts, but no differences in lymphatic tissue weights (Figure 2A). In submissive female mice, a tendency for reduced thymus weights as compared to dominant mice was observed (*p* = 0.1, Figure 2A) but no further differences in body or spleen weight. To investigate the stress level of dominant and submissive mice, we weighed adrenal glands and measured CORT via HPLC analysis after extraction from serum samples (Figures 2B,C). Changes in adrenal glands weight were not observed for male mice, but increased CORT levels in dominant males, indicating a higher stress level. Interestingly, female mice showed precisely the opposite: submissive animals seemed to be more stressed, as illustrated by increased adrenal glands weight and elevated CORT levels as compared to dominant female mice.

### Whole-Brain Transcriptomics Analysis Comparing Dominant and Submissive Mice

**FIGURE 3:**
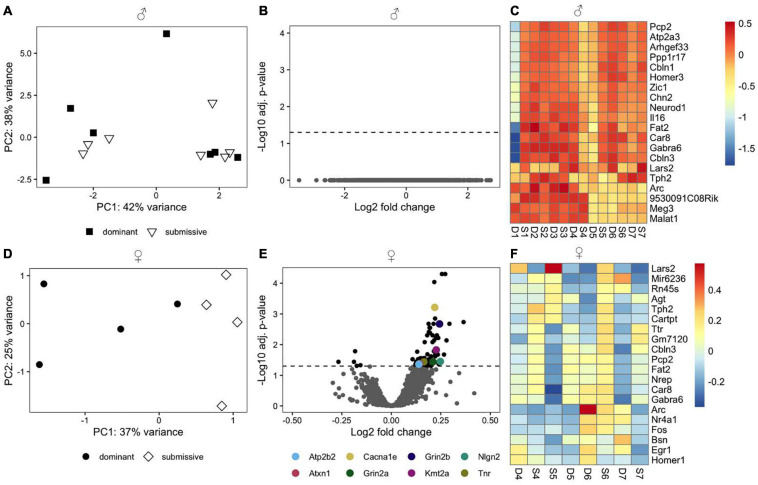
Transcriptomic profile of brain samples from male and female mice according to social rank. Differences in the multivariate expression pattern in brain hemispheres of male **(A)** and female **(D)** dominant and submissive mice were visualized with PCA using the top 500 most variable genes. Volcano plots display differentially expressed genes (adjusted *p* < 0.05, shown as black dots, *n* = 7 for males and 4 for females) in male **(B)** or female **(E)** mice. Positive log2 fold changes indicate genes that are up-regulated in dominant mice, while negative log2 fold changes correspond to down-regulated genes. The horizontal dashed line on the volcano plots labels the threshold for differentially expressed genes (adjusted *p* < 0.05). The 20 genes with the highest variance across samples were determined for male **(C)** and female **(F)** animals and visualized with a heatmap using rlog transformed counts. Counts were centered around the respective mean value for each gene.

We employed RNA-seq in order to investigate changes in brain transcriptome profiles in male and female mice related to dominant or submissive behavior. PCA using the top 500 most variable genes did not reveal a discernible separation of male animals according to their social status (Figure 3A). Additionally, we did not identify any genes that passed the criteria for differential expression (Figure 3B). Interestingly, several genes predominantly expressed in the cerebellum, including Pcp2, Atp2a3, Ppp1r17, Cbln1, and Homer3, exhibited a very similar expression pattern. These were among the top 20 most variable genes (Figure 3C). However, the difference between experimental conditions was not statistically significant.

Principal component analysis using the top 500 most variable genes in females revealed a clear separation between dominant and submissive animals (Figure 3D). In the right brain hemispheres of dominant female mice, 74 DEGs were up-regulated relative to their submissive counterparts, while five genes were down-regulated (Figure 3E and Supplementary Table 1). Importantly, several genes that might be involved in controlling hyperexcitability in different brain regions and thereby modulating behavioral fear response were upregulated in dominant females as compared to submissive mice. These include the Ca^2+^ voltage-gated R type channel subunit alpha 1E gene (Cacna1e), the glycoprotein tenascin-R (Tnr) which is part of the extracellular matrix in the CNS (Weber et al., 1999) as well as the adhesion molecule neuroligin-2 (Nlg2) which is selectively expressed at inhibitory synapses (Graf et al., 2004). The glutamate transporter gene Slc1a2 was also associated with significantly higher expression in dominant female mice’s brains. Furthermore, Grin2a and Grin2b, two major subunits of N-methyl-D aspartate (NMDA) receptors that are involved in synaptic plasticity (Kiyama et al., 1998; Zhao et al., 2005) were both downregulated in submissive mice. Two additional genes that exhibited significantly higher expression in female dominant animals are the plasma membrane calcium-transporting ATPase 2 (Atp2b2) and ataxin-1 (Atxn1). These genes are involved in cerebellum development, and genetic ablation or mutations are associated with neurodegeneration, impaired locomotion, and ataxia (Kozel et al., 1998; Matilla et al., 1998). The methyltransferase gene Kmt2a, which is implicated in short term-synaptic plasticity (Jakovcevski et al., 2015) was also upregulated in dominant female mice (Figure 3E). The top 20 most variable genes in female mice are shown in Figure 3F.

### m^6^A-RNA Modification Is Differentially Affected in Dominant and Submissive Mice of Male and Female Sex

**FIGURE 4:**
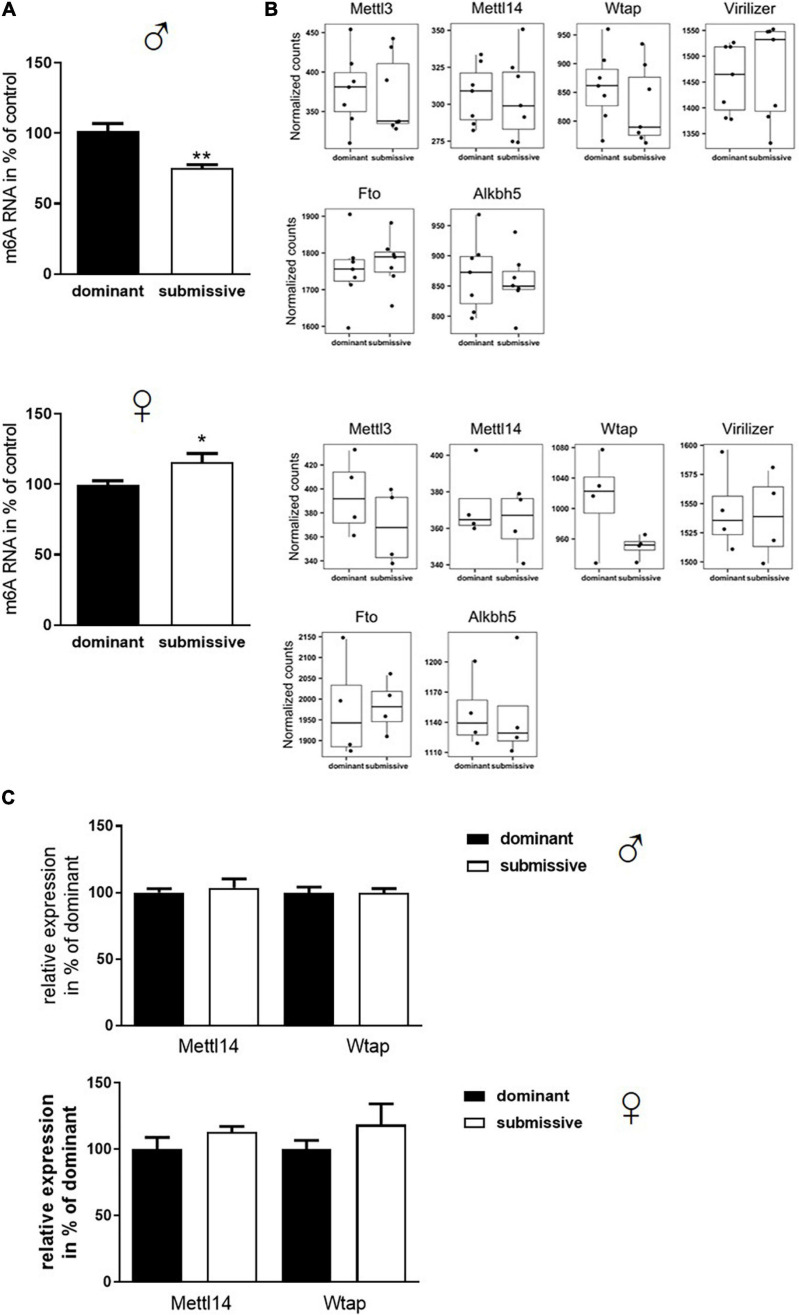
Changes in m^6^A mRNA modification in relation to stress evoked by social rank. Isolated mRNA was applied to measure m^6^A modification (**A**, *n* = 7 for dominant; *n* = 6 for submissive groups). Data are normalized to the mean value of dominant animals (set to 100%) and presented as mean + SEM. Statistical analysis was performed with Student’s *t*-test; (**p < 0.01; *p < 0.05). Specificity of the used kit [EpiQuik m6A RNA Methylation Quantification Kit (Colorimetric), EpiGentek] was confirmed by utilizing RNA from a Mettl3 knock-out *Drosophila* mutant (see Supplementary Figure 1). **(B)** Normalized transcript counts of genes associated with m^6^A modification (*n* = 7 per group for males; *n* = 4 per group for females). Data are represented as boxplots showing the 1st quartile, median, 3rd quartile and interquartile range and as individual values. **(C)** RNA levels of Mettl14 and Wtap were assessed via qPCR (*n* = 4–6 per group) and normalized to GAPDH mRNA levels. Data are shown as mean + SEM.

Transcriptomic analysis of brain mRNA revealed only minor differences in gene expression in female mice of different social hierarchy stage, but no changes between dominant and submissive male mice. Nevertheless, not only transcriptional activity regulates gene expression but also modification of RNA greatly affects half-life time and accessibility (Wang et al., 2014; Mauer et al., 2017). In the groups investigated here, N^6^-adenosyl-mRNA-methylation was decreased in submissive males (25%) but increased in submissive females (15%) as compared to their respective counterparts (Figure 4A). m^6^A is the most prominent type of mRNA modification (Wei et al., 1975; Dominissini et al., 2012; Meyer et al., 2012) which is conducted by a complex, including METTL3 and METTL14 methyltransferases, whereby METTL3 provides the catalytic subunit and METTL14 the target recognition unit (Boker et al., 1997; Liu et al., 2014; Schwartz et al., 2014; Wang et al., 2014). WTAP operates as a regulatory subunit, and Virilizer is required for methyltransferase activity (Liu et al., 2014; Ping et al., 2014; Schwartz et al., 2014). The methylation can be reversed by activation of demethylases FTO and ALKBH5 (Jia et al., 2011; Fu et al., 2013; Zheng et al., 2013). Even though we did not find significant differences in the expression of the genes encoding for the m^6^A enzymatic complex in our transcriptomic analysis before, we visualized and inspected the normalized RNA counts of all involved players. However, we could not detect any non-significant trends – neither for males nor for females – regarding the respective transcript levels in relation to social positioning (Figure 4B). To verify the findings from the transcriptomics analysis with a distinct method, Mettl14 and Wtap mRNA levels were determined by qPCR (Figure 4C) which confirmed the unaltered expression. A slight increase in expression of both mRNAs could be observed for submissive females as compared to their dominant counterparts. However, this did not reach statistical significance (*p* < 0.3 for both comparisons).

**FIGURE 5:**
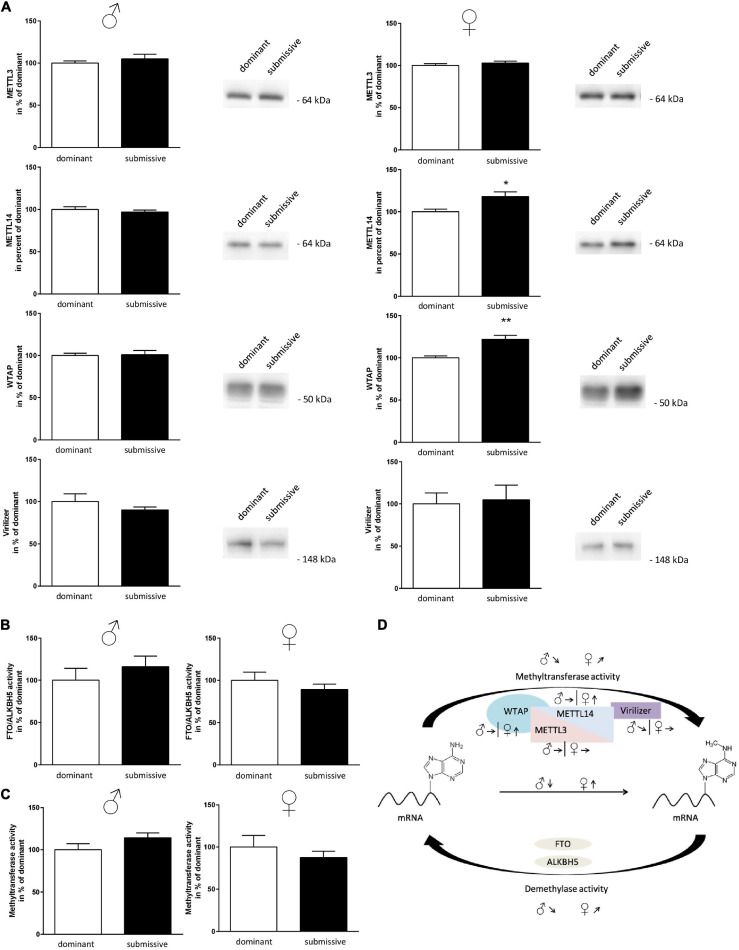
Changes in amount or activity of enzymes involved in m^6^A modification. Proteins of nuclei extracts derived from brain hemispheres of dominant and submissive mice were separated on polyacrylamide gels, transferred to nitrocellulose and METTL3, METTL14, WTAP, and Virilizer protein amounts were analyzed by using specific antibodies and a chemiluminescent detection system. 20 μg of protein per lane were subjected to the gel **(A)** and equal loading controlled by Ponceau S red staining of the membrane (data not shown). FTO/ALKBH5 demethylase **(B)** and methyltransferase activity **(C)** of nuclei extracts was determined by using the respective enzyme assay. Data are normalized to the mean value of dominant animals (set to 100%) and presented as mean + SEM. Statistical analysis was performed with Student’s *t*-test; (**p < 0.01; *p < 0.05, *n* = 7 per group for males, *n* = 4 per group for females). **(D)** Graphical summary of changes in the amounts of METTL3/METTL14-complex proteins and methyltransferase or demethylase activity for male and female mice. Arrows indicate the comparisons of the submissive animals with their dominant counterparts [↑, increase; ↓, decrease; ↗,↘, trends; Figure was modified according to Deng et al. (2018)].

Subsequently, as mRNA levels of all complex participants could not explain the observation of altered m^6^A levels, protein amount and enzymatic activity of enzymes involved in m^6^A modification were analyzed. In male mice, no differences in protein amounts of METTL3, METTL14, and WTAP were identified in whole brain hemispheres of dominant as compared to submissive individuals. Compared to dominant mice, only a tendency toward a reduced amount of Virilizer was determined in submissive male mice, which was, however, not statistically significant (*p* = 0.1, Figure 5A). Although no differences between METTL3 and Virilizer were observed in the investigated groups in female mice, METTL14 and WTAP showed significantly increased values in submissive females that might explain the previously measured increase of m^6^A mRNA (Figures 4A, 5A). In addition, the activity of the demethylases FTO and ALKBH5 was investigated. Submissive males and dominant females tended to demonstrate a higher activity of the investigated demethylases, but differences did not reach statistical significance (Figure 5B, *p* = 0.4 for both).

Furthermore, methyltransferase activity was investigated using a luciferase-based assay. The assay was designed to detect the activity of any methyltransferases within the respective sample. Therefore, we aimed at gaining at least partial specificity toward m^6^A methyltransferases by using polyA RNA as substrate. Sinefungin, a non-specific inhibitor of m^6^A methyltransferase, was used to test this at a concentration where mainly METTL3/METTL14 should be affected (10 μM) (Selberg et al., 2019). About 51% of the signal derived by methyltransferase activity was inhibited by Sinefungin, suggesting that at least a large part of the reaction was based on METTL3/METTL14 complex activity (see Supplementary Figure 3). Contrary to our expectations, the measured methyltransferase activity tended to be directed in the opposite direction of the measured m^6^A methylation levels: higher activity in submissive males and dominant females as compared to their respective counterparts were detected, while not being statistically significant (Figure 5C, *p* = 0.1 for males and *p* = 0.4 for females). For a summary of observed changes in regard to m^6^A modification see Figure 5D.

## Discussion

The tube test is a well-established paradigm to determine the social cage hierarchy of group-housed mice (Lindzey et al., 1961; Yang et al., 2015). Successful performance of the tube test in our study, and thereby stratification of hierarchical groups, was shown by the significantly lower number of wins of the submissive animals. The key finding of the comparison of dominant and submissive mice is the difference in m^6^A methylation of brain parenchymal tissue, which interestingly occurred in a gender-specific manner.

The cage hierarchy of rodents such as mice depends on intrinsic mechanisms, previous experiences, and hormone levels such as testosterone (van den Berg et al., 2015; Williamson et al., 2017). Male mice kept in groups exhibit a linear, stable cage hierarchy (van den Berg et al., 2015; Williamson et al., 2016), suggesting high sociocognitive competence, which is consistent with our results. Williamson et al. (2019) also investigated social hierarchies in female mice housed in groups of twelve. Their results indicate that alpha female mice do not show dominance over all cage mates. For our female groups consisting of only four mice each, dominant and submissive individuals were well defined, but intermediates were not distinguishable. This suggests a non-linear hierarchy as the main difference to male animals and is in accordance with the findings from larger female groups.

Dominance in male mice has been shown to be indirectly proportional to body weight: elevated body weight leads to submissive behavior, for example, in CD-1 mice (Kim et al., 2015). Similarly, in our study, submissive male mice displayed a significant increase in body weight compared to their dominant counterparts. Further dominance measures were not performed, however, a good correlation of the tube test with such has been described before for the C57BL/6J strain (Fan et al., 2019). These reports, however, mainly include male mice and sub-strain specificity might not have always been clearly described.

The intrinsic social status of the mice influenced their stress levels: submissive males presented lower serum corticosterone amounts than dominant males, which is in accordance with some reports from literature (Williamson et al., 2017). Pletzer et al. (2007), for example, described that the lowest corticosterone level was observed in active subordinate male Balb-C mice (depending on the coping style). However, it has to be mentioned that observations concerning corticosterone levels are contradictory as also contrary reports exist (Meehan et al., 1987; Fano et al., 2001) or reports revealing indistinguishable corticosterone levels (Hilakivi et al., 1989). This might be due to the measurement technique, used strain or sub-strain or even to how the tested groups were established. E.g., it has been shown that mice reared in a group of siblings since weaning showed unaltered basal corticosterone levels (Bartolomucci et al., 2001). Contrary to the reduced corticosterone level for the male mice tested in our study, female submissive mice displayed higher adrenal gland weights and corticosterone serum levels than their dominant counterparts, which is also described in previously published data (Williamson et al., 2019). This observation confirms that social status causes gender-specific stress in the mice.

After identifying dominant and submissive individuals, whole brain transcriptomic analysis was conducted to analyze gene regulation regarding social hierarchy status. Significant differences between dominant male mice and submissive ones were not detected and only subtle changes were observed between the investigated groups of female mice. Amongst the most variable genes cerebellar genes occurred such as Pcp2. Even if the observed differences did not reach statistical significance, it is of note that corticosterone injections are able to affect motor coordination in mice (Harle et al., 2017) and that social defeat elicits motor impairment (Tomas-Roig et al., 2016).

Especially in dominant female mice compared to their submissive counterparts, genes involved in modulating fear response and anxiety (e.g., Cacna1e, Nlg2, Tnr, Grin2a, and Grin2b) were up-regulated. For example, Nlg2 was increased by 1.2 fold in dominant females. Nlg2, a cell adhesion molecule enriched in the post-synapses of inhibitory neurons [GABAergic synapses, (Graf et al., 2004)], is known to be associated with inhibitory synaptic transmission. Nlg2 knock-out mice showed, for instance, anxiety-like behavior as indicated by significantly less time spent in the center of the arena in an open field test while spending considerably more time in the dark compartment in the light/dark test (Blundell et al., 2009). As another example, Tenascin R (Tnr) – a glycoprotein that is a component of the extracellular matrix in the CNS – was up-regulated 1.1 fold in dominant females compared to submissive ones. This protein plays a role in synaptic plasticity by modulating long-term potentiation. Tnr-deficient mice exhibited reduced exploration time and time spent in the center of an open field test (Freitag et al., 2003). Moreover, Tnr knock-out mice were associated with an anxiety-like behavior, illustrated by less time spent in the open arms of an elevated plus-maze test (Freitag et al., 2003). This phenotype might potentially be explained by impaired inhibitory control in the hippocampus and prefrontal cortex as a result of Tnr ablation, leading to enhanced excitability indicated by altered neural network oscillations and increased amplitudes of evoked potentials (Gurevicius et al., 2004). As demonstrated by these two examples, it can be assumed that dominant females behave less anxiously than submissive individuals. However, our study is limited by the fact that no anxiety-associated or depression-like behavior was assessed. Therefore, we cannot undoubtedly demonstrate if this holds true. For male mice, however, submissive animals identified by the tube test displayed a higher preference for the short acting benzodiazepine midazolam, resembling high abuse liability in humans suffering from anxiety (Riad-Allen and van der Kooy, 2013). Moreover, the antidepressant fluoxetine could occlude submissive behavior, evoked by chronic restraint stress in male mice (Park et al., 2018). Other reports come to the conclusion that social status does not correlate with altered anxiety behavior as, e.g., Pallé et al. (2019) found no differences in the elevated plus maze in male C57BL/6J mice. This was confirmed by another study which described that subordinate mice had a significantly lower SI ratio than dominant mice (Sabanovic et al., 2020). This might also have been evoked by the chronic social defeat stress that the mice experienced 2 weeks before hierarchy assessment. For female mice, data are scarce. Nevertheless, the assumption that dominant females might be less anxious may be further supported by the reduced CORT serum levels in dominant females compared to submissive animals found in our study, considering that corticosterone levels have already been defined to directly relate to anxiety-like behavior (McGill et al., 2006; Chaves et al., 2019). Since in our case, the transcriptomic analysis was performed on whole brain hemispheres, the DEGs cannot be traced back to relevant functional regions. This could explain the rather small effect sizes of differential expression observed in females or be the reason for the lack of differences in the males when comparing dominant and submissive individuals. Brain regions associated with depression, evoked, e.g., by chronic social defeat stress (Colyn et al., 2019; Zhang et al., 2019; Heshmati et al., 2020), comprise the nucleus accumbens, prefrontal cortex, amygdala, and ventral hippocampus. Therefore, it is not astonishing, that in these discrete areas, more DEGs are eminent such as, for example, genes encoding olfactory and vomeronasal receptors in the medial prefrontal cortex (Pallé et al., 2019).

In contrast to the lack of obvious global effects on the transcriptomic level, m^6^A mRNA methylation was affected in isolates from whole brain hemispheres in both sexes: higher methylation status was measured in dominant males and submissive females. m^6^A is influencing lifespan, processing, and translation of mRNA (Liu and Pan, 2016; Maity and Das, 2016). Interestingly, m^6^A abundance has already been associated with corticosterone levels in another context; namely, it has been shown that physical stress influences m^6^A mRNA methylation (Engel et al., 2018). The mice used for these experiments were exposed to 15 min restraint stress, resulting in a region-dependent m^6^A regulation. Increased methylation status occurred in the central amygdala, but a decreased amount of m^6^A was observed in the medial prefrontal cortex. Physical stress induces a significant rise in corticosterone levels even after a single exposure, while experienced emotional and social stress occurring one time only does not influence these levels (Kavushansky et al., 2009; Nakatake et al., 2020). In our study, mice displayed social hierarchy state-dependent increased stress levels, as shown by secreted corticosterone. Consequently, repeatedly experienced social stress seems to be more comparable to physical stress application in this regard.

To decipher the molecular mechanisms leading to the observed altered m^6^A mRNA methylation status, we examined proteins involved in m^6^A modification with respect to their amount and activity in the investigated groups. The increase of m^6^A in brain samples of submissive females was supported by elevated protein levels of METTL14 and WTAP. METTL14 is assumed to act as an enhancer for the activity of METTL3 in the methylation complex (Wang et al., 2016; Zhou and Pan, 2016). Therefore, an increased expression of METTL14 should support METTL3 activity, the main catalytic subunit of the METTL3/METTL14 protein complex. In male mice, protein levels did not differ between the investigated groups. It would have been reasonable to assume that the altered methylation patterns were due to increased activity of the responsible methyltransferase in dominant males and submissive females. However, neither for males nor for females differences in methyltransferase activity were detected under these conditions. Unfortunately, there is currently – to our knowledge – no selective assay to measure the specific activity of the METTL3/METTL14 protein complex. Therefore, we utilized an assay, which measures the activity of methyltransferases rather non-specifically. Although the applied conditions should prioritize the METTL3/METTL14 complex, we cannot exclude that the activity of other methyltransferases overcast this RNA-specific activity.

Future studies should focus on a detailed investigation of stress-related areas such as the amygdala and ventral hippocampus with respect to m^6^A methylation and the related enzymatic machinery to understand the gender-specific differences of altered m^6^A methylation patterns in the context of social stress. Additionally, the role of sex hormones has to be investigated. At least in zebra fish, Mettl3, and m^6^A modification have been shown to be involved in gamete maturation (Xia et al., 2018). This might indicate that sex hormones also affect amount and/or activity of the methylation status via the involved enzymatic complexes. For example, chorionic gonadotropin decreased Mettl14 by reducing its stability in Leydig cells (Chen et al., 2021). If such regulation is also occurring in the brain and how a potential sex hormone-dependency is conferred has not been elucidated yet. Moreover, the identification of the targeted mRNAs and the distinct role of glucocorticoid signaling will lead to a better understanding of sex-dependent stress-reactivity. Activators of the METTL3/WTAP complex have already been identified by an *in silico* discovery strategy (Selberg et al., 2019). Analogous approaches for inhibitors together with target mRNA identification will probably open up new avenues for epitranscriptomics-based treatment of stress-related disorders. In this regard, investigating also intermediate animals identified by the tube test and trying to shift their position by interference with the methylation apparatus, can be a future approach to further contribute to our understanding of coping with social stress.

## Data Availability Statement

The raw sequencing data from this study are available at GEO under the accession number GSE161198 (https://www.ncbi.nlm.nih.gov/geo/query/acc.cgi?acc=GSE161198).

## Ethics Statement

The animal study was reviewed and approved by the Landesuntersuchungsamt Rhineland-Palatinate.

## Author Contributions

KE and SG contributed to conception and design of the study. MdSG, ST, VTTN and TT conducted the experiments. MdSG and HT designed the figures and drafted the manuscript. HT and TT performed the analysis of sequencing data. All authors contributed to manuscript revision, read, and approved the submitted version.

## Conflict of Interest

The authors declare that the research was conducted in the absence of any commercial or financial relationships that could be construed as a potential conflict of interest.

## Publisher’s Note

All claims expressed in this article are solely those of the authors and do not necessarily represent those of their affiliated organizations, or those of the publisher, the editors and the reviewers. Any product that may be evaluated in this article, or claim that may be made by its manufacturer, is not guaranteed or endorsed by the publisher.

## Supplementary Material

Supplementary Figure 1 Specificity of m6A mRNA modification measurement.

Supplementary Figure 2 Uncropped Western blot image example.

Supplementary Figure 3 Methyltransferase activity assay with Sinefungin as inhibitor.

Supplementary Table 1 Differentially expressed genes in the right brain hemispheres of dominant versus submissive female mice.
