# Supplementary material for: N6 -Methyladenosine Modification in Chronic Stress Response Due to Social Hierarchy Positioning of Mice
Source: Front Cell Dev Biol. 2021 Aug 20;9:705986. doi: 10.3389/fcell.2021.705986 (PMC8417747; doi:10.3389/fcell.2021.705986)
Supplement: Supplementary Figure 1 — Specificity of m6A mRNA modification measurement. [file Data_Sheet_1.DOCX]

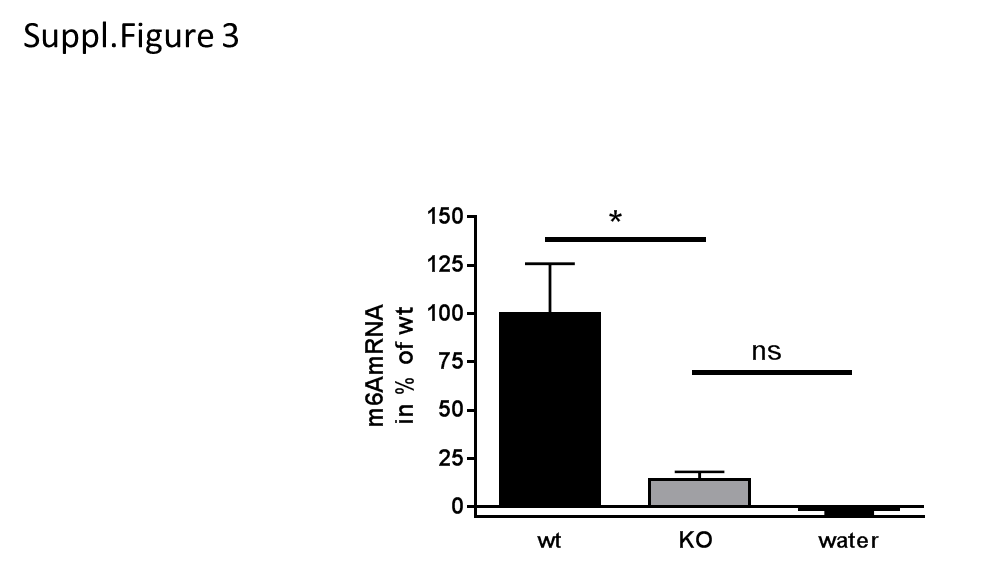


**Supplementary Figure 1: Specificity of m^6^A mRNA modification measurement**

To evaluate the specificity of the antibody-based assay that was used to quantify m^6^A modification in murine mRNA, pooled RNA of wild type flies (*D. melanogaster*) and flies with Mettl3 knock-out was used. 200ng of RNA was subjected to the assay in triplicate, water served as absolute negative control. Deletion of the methyltransferase resulted in a significant reduction of the signal (14% of wild type value) that was indifferent to water control.
