# Supplementary material for: N6 -Methyladenosine Modification in Chronic Stress Response Due to Social Hierarchy Positioning of Mice
Source: Front Cell Dev Biol. 2021 Aug 20;9:705986. doi: 10.3389/fcell.2021.705986 (PMC8417747; doi:10.3389/fcell.2021.705986)
Supplement: Supplementary Figure 2 — Uncropped Western blot image example. [file Data_Sheet_2.DOCX]

**
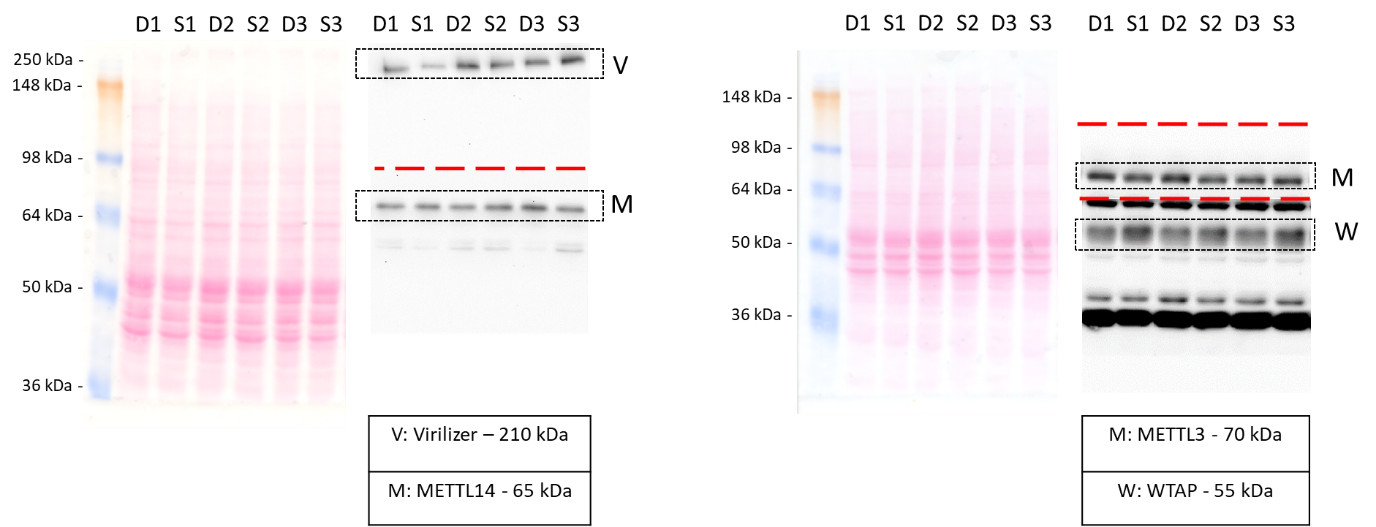
**

**Supplementary Figure 2: Uncropped Western blot image example**

Nuclear fractions of left brain hemispheres were extracted and protein concentration adjusted to 20 μg/10 μl. Proteins were separated on a 10% SDS-polyacrylamide gel and equal loading confirmed by Ponceau S red staining after transfer on nitrocellulose membranes. After destaining with PBS, membranes were cut in distinct pieces (cutting positions indicated as red dashed lines) by using the molecular weight marker (SeeBlue™ Plus2 Pre-stained Protein Standard, ThermoFisherScientific) and subjected accordingly to immune-detection of either protein. An exemplary blot with three samples per group (d= dominant, s= submissive) is shown (right: samples from male animals; right: samples from female animals).
