# Supplementary material for: N6 -Methyladenosine Modification in Chronic Stress Response Due to Social Hierarchy Positioning of Mice
Source: Front Cell Dev Biol. 2021 Aug 20;9:705986. doi: 10.3389/fcell.2021.705986 (PMC8417747; doi:10.3389/fcell.2021.705986)
Supplement: Supplementary Figure 3 — Methyltransferase activity assay with Sinefungin as inhibitor. [file Data_Sheet_3.DOCX]

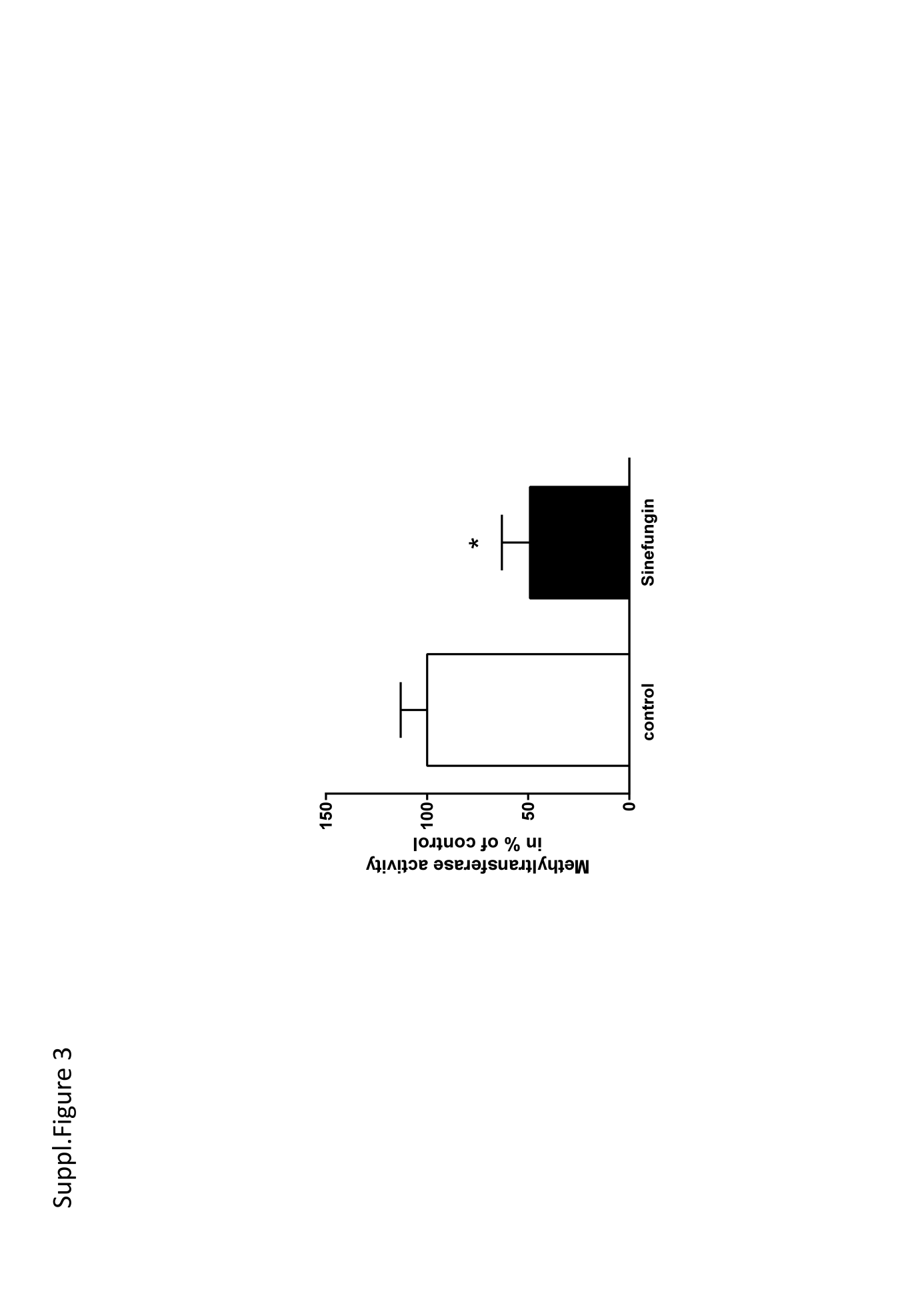


**Supplementary Figure 3: Methyltransferase activity assay with Sinefungin as inhibitor**

Nuclei extract (5 μg protein per sample) was subjected to the luciferase-based methyltransferase assay. Samples without SAM served as background control. Samples with 10 μM Sinefungin revealed a reduced (-51%, p=0.02) activity, indicating the participation of the METTL3/METTL14 complex. Data are presented as mean + SEM. Statistical analysis was performed with Student’s t-test; *, p<0.05, n=7 per measurement condition).
