# Supplementary material for: N6 -Methyladenosine Modification in Chronic Stress Response Due to Social Hierarchy Positioning of Mice
Source: Front Cell Dev Biol. 2021 Aug 20;9:705986. doi: 10.3389/fcell.2021.705986 (PMC8417747; doi:10.3389/fcell.2021.705986)
Supplement: Supplementary Table 1 — Differentially expressed genes in the right brain hemispheres of dominant versus submissive female mice. [file Table_1.DOCX]

**Supplementary Table 1: Differentially expressed genes in the right brain hemispheres of dominant versus submissive female mice.** Positive log2 fold changes indicate genes that are up-regulated in dominant mice, while negative log2 fold changes correspond to down-regulated genes.

|  | **Base mean** | **Log2 fold change** | **Standard error** | **Raw p-value** | **Adjusted p-value** |
| --- | --- | --- | --- | --- | --- |
| R3hdm1 | 2898.833371 | 0.255598288 | 0.044602 | 1E-08 | 5.04E-05 |
| Pclo | 5737.759583 | 0.272267176 | 0.048412 | 1.87E-08 | 5.04E-05 |
| Xist | 5633.815941 | 0.217072869 | 0.039844 | 5.09E-08 | 9.16E-05 |
| Cacna1e | 4690.128043 | 0.219065184 | 0.043422 | 4.54E-07 | 0.000612 |
| Mkl2 | 3325.672472 | 0.221018542 | 0.045676 | 1.31E-06 | 0.00141 |
| Inf2 | 699.2154385 | 0.364717807 | 0.076805 | 2.05E-06 | 0.001843 |
| Nav1 | 900.8426188 | 0.293553699 | 0.062863 | 3.02E-06 | 0.002092 |
| Arhgap32 | 6345.6523 | 0.1882212 | 0.040432 | 3.24E-06 | 0.002092 |
| AI593442 | 2505.566689 | 0.213111494 | 0.045931 | 3.49E-06 | 0.002092 |
| Grin2b | 3797.876255 | 0.243467499 | 0.052747 | 3.92E-06 | 0.002115 |
| Snhg11 | 8672.171077 | 0.195696936 | 0.043322 | 6.27E-06 | 0.002819 |
| Kcnb1 | 3030.687494 | 0.193304376 | 0.042621 | 5.75E-06 | 0.002819 |
| Sipa1l1 | 3522.208459 | 0.189571162 | 0.042759 | 9.27E-06 | 0.003852 |
| Ryr3 | 1820.867694 | 0.195068817 | 0.044309 | 1.07E-05 | 0.004128 |
| Atp2b4 | 2853.241511 | 0.207780325 | 0.048045 | 1.53E-05 | 0.004966 |
| Srrm2 | 3665.863457 | 0.181206928 | 0.04195 | 1.56E-05 | 0.004966 |
| Ago3 | 1068.96475 | 0.230052122 | 0.053109 | 1.48E-05 | 0.004966 |
| R3hdm2 | 1053.86264 | 0.237177031 | 0.055794 | 2.13E-05 | 0.006051 |
| Gm15800 | 4351.325227 | 0.224930299 | 0.052829 | 2.07E-05 | 0.006051 |
| Wasf1 | 825.3974282 | 0.278707991 | 0.066415 | 2.71E-05 | 0.007272 |
| Celf2 | 3298.768448 | 0.215974343 | 0.051583 | 2.83E-05 | 0.007272 |
| Nfasc | 2546.678074 | 0.168534195 | 0.040744 | 3.53E-05 | 0.008281 |
| D430041D05Rik | 2256.244853 | 0.208122196 | 0.050234 | 3.43E-05 | 0.008281 |
|  | **Base mean** | **Log2 fold change** | **Standard error** | **Raw p-value** | **Adjusted p-value** |
| Kmt2a | 1583.443677 | 0.226801151 | 0.056834 | 6.59E-05 | 0.014829 |
| Crebbp | 1248.5452 | 0.214841038 | 0.054416 | 7.88E-05 | 0.016362 |
| Wdr6 | 3178.566443 | -0.183026349 | 0.046322 | 7.78E-05 | 0.016362 |
| Gas7 | 7513.002938 | 0.130623034 | 0.033236 | 8.49E-05 | 0.016974 |
| Rorb | 1297.779919 | 0.182202578 | 0.047028 | 0.000107 | 0.02062 |
| Syngap1 | 1173.777285 | 0.266256743 | 0.069042 | 0.000115 | 0.021024 |
| Mical3 | 1023.445003 | 0.221722515 | 0.057669 | 0.000121 | 0.021024 |
| Syn1 | 1372.2653 | 0.235033867 | 0.061119 | 0.00012 | 0.021024 |
| Cobl | 1122.348566 | 0.201134314 | 0.052985 | 0.000147 | 0.024057 |
| Nfat5 | 1162.547262 | 0.221548567 | 0.058334 | 0.000146 | 0.024057 |
| Meg3 | 17622.00185 | 0.166586184 | 0.044317 | 0.000171 | 0.027094 |
| Hivep2 | 5855.26203 | 0.152019444 | 0.040581 | 0.00018 | 0.027714 |
| Fubp1 | 2576.518146 | 0.145835832 | 0.039008 | 0.000185 | 0.027759 |
| Slc1a2 | 38773.4549 | 0.107179423 | 0.028967 | 0.000216 | 0.02984 |
| Cttnbp2 | 1594.620891 | 0.18907601 | 0.050932 | 0.000205 | 0.02984 |
| Wnk1 | 2812.801197 | 0.160557482 | 0.043338 | 0.000212 | 0.02984 |
| Ank3 | 4668.595308 | 0.157814022 | 0.042788 | 0.000226 | 0.029898 |
| Smg1 | 4550.05974 | 0.134757985 | 0.036551 | 0.000227 | 0.029898 |
| Nlgn2 | 1032.589465 | 0.247544621 | 0.068034 | 0.000274 | 0.035251 |
| Ypel2 | 1409.372869 | 0.164066249 | 0.045213 | 0.000285 | 0.035761 |
| Raph1 | 852.7197486 | 0.220408115 | 0.061712 | 0.000355 | 0.036191 |
| Tnr | 1658.315594 | 0.164440645 | 0.045717 | 0.000322 | 0.036191 |
| Arfgef3 | 2156.062724 | 0.147328958 | 0.04153 | 0.000389 | 0.036191 |
| Tnrc6b | 2160.285226 | 0.158777352 | 0.044813 | 0.000395 | 0.036191 |
| Lrrk2 | 2580.626141 | 0.179891293 | 0.050233 | 0.000342 | 0.036191 |
|  | **Base mean** | **Log2 fold change** | **Standard error** | **Raw p-value** | **Adjusted p-value** |
| Grin2a | 1463.197861 | 0.210298636 | 0.05819 | 0.000301 | 0.036191 |
| Pde10a | 4062.018902 | 0.188002917 | 0.052857 | 0.000375 | 0.036191 |
| Rasgrp1 | 7659.160958 | 0.15049047 | 0.042467 | 0.000395 | 0.036191 |
| Kcnq2 | 4138.015934 | 0.141906582 | 0.039366 | 0.000312 | 0.036191 |
| Setd7 | 3141.228502 | 0.142423102 | 0.040171 | 0.000392 | 0.036191 |
| Insig1 | 1535.373769 | -0.188719458 | 0.053153 | 0.000384 | 0.036191 |
| Wdfy3 | 3685.7063 | 0.149968755 | 0.041843 | 0.000338 | 0.036191 |
| Thsd7a | 1566.703035 | 0.175117208 | 0.049246 | 0.000377 | 0.036191 |
| Aak1 | 5851.323703 | 0.125436679 | 0.034709 | 0.000302 | 0.036191 |
| Mical2 | 2811.32653 | 0.186666092 | 0.052658 | 0.000393 | 0.036191 |
| Rbm3 | 885.0099161 | -0.267186691 | 0.074861 | 0.000358 | 0.036191 |
| Mir124a-1hg | 1174.800988 | 0.239615978 | 0.067817 | 0.00041 | 0.036942 |
| Opcml | 3831.287494 | 0.139685617 | 0.039831 | 0.000453 | 0.040131 |
| Atxn1 | 2907.486547 | 0.142650035 | 0.040818 | 0.000474 | 0.04132 |
| Tanc2 | 3226.530311 | 0.179908194 | 0.051648 | 0.000495 | 0.042448 |
| Cx3cl1 | 5789.721874 | 0.133287517 | 0.038317 | 0.000504 | 0.04254 |
| Astn1 | 4043.691147 | 0.122040101 | 0.035186 | 0.000524 | 0.043156 |
| Atp2b2 | 9835.706063 | 0.138820714 | 0.040094 | 0.000535 | 0.043156 |
| Sorbs2 | 2443.162155 | 0.177321738 | 0.051211 | 0.000535 | 0.043156 |
| Pcdh1 | 878.8545788 | 0.239452536 | 0.06924 | 0.000544 | 0.043167 |
| Nrxn3 | 4580.697542 | 0.124567863 | 0.036184 | 0.000576 | 0.043612 |
| Kcnma1 | 3163.262557 | 0.143946356 | 0.041756 | 0.000566 | 0.043612 |
| Asap1 | 1438.703652 | 0.162046774 | 0.047105 | 0.000581 | 0.043612 |
| Cacna1c | 1407.049818 | 0.164728843 | 0.04788 | 0.000581 | 0.043612 |
| Srcin1 | 1590.488941 | 0.169518236 | 0.049619 | 0.000635 | 0.046556 |
|  | **Base mean** | **Log2 fold change** | **Standard error** | **Raw p-value** | **Adjusted p-value** |
| Prkce | 4043.769905 | 0.141748674 | 0.041509 | 0.000638 | 0.046556 |
| 1110004F10Rik | 1291.642586 | -0.156050865 | 0.045749 | 0.000647 | 0.046596 |
| Vamp2 | 2505.543565 | 0.17454322 | 0.051343 | 0.000675 | 0.04733 |
| Dio2 | 1851.592321 | 0.18153832 | 0.053369 | 0.00067 | 0.04733 |
| D630045J12Rik | 759.7396127 | 0.200459205 | 0.059146 | 0.000701 | 0.04852 |
| Sparc | 5917.830477 | -0.174794084 | 0.051667 | 0.000717 | 0.048998 |
